# Pills of Iodide of Iron

**Published:** 1845-04

**Authors:** 


*Pills of Iodide of Iron.*—Dr. Christison recommends the following simple method of preparing these pills, which was communicated to him by Mr. Robert Leslie, late apothecary in the Royal Infirmary of Glasgow. Take of iodine 127 grs., iron wire about the thickness of a thin quill, half an ounce, distilled water 75 minims. Agitate them briskly together in a strong ounce phial, provided with a well-fitted glass stopper, until the froth which forms becomes white, which will happen in less than ten minutes. Pour the liquid upon two drachms of finely-powdered loaf-sugar in a little mortar, and triturate immediately and briskly for a few minutes; add gradually a mixture of the following powders, viz: liquorice powder half an ounce, powder of gum arabic a drachm and a half, and flour one drachm. Divide the mass into 144 pills.

Each pill contains about a grain of iodide of iron. The only alteration I have made in Mr. Leslie's formula, is the substitution of coarse for fine iron wire; first, because less heat is produced; and secondly, because the resulting solution is more easily poured off.

In operations on the large scale, the bottle ought to be wrapped in a strong towel, in case of an explosion being caused by the evolution of steam from the heat produced; and even on the small scale, the stopper must be held firmly, otherwise it will probably be blown out, and the materials lost.

After all that has been recently written on the action of sugar in preventing the proto-salts of iron from alteration under exposure to the air, it is unnecessary to point out the reason why these pills keep remarkably well. With the *syrupus iodidi ferri* of the last Edinburgh Pharmacopœia, and this *pilula iodidi ferri*, it appears to me that every other pharceutic form of the salt may be dispensed with.—*Pharm. Trans.*

